# Accurate Heart Rate and Respiration Rate Detection Based on a Higher-Order Harmonics Peak Selection Method Using Radar Non-Contact Sensors

**DOI:** 10.3390/s22010083

**Published:** 2021-12-23

**Authors:** Hongqiang Xu, Malikeh P. Ebrahim, Kareeb Hasan, Fatemeh Heydari, Paul Howley, Mehmet Rasit Yuce

**Affiliations:** 1 Department of Electrical and Computer Systems Engineering, Monash University, Clayton, VIC 3800, Australia; charlie.xu@monash.edu (H.X.); melika.pour.ebrahim@monash.edu (M.P.E.); kareeb.hasan2@monash.edu (K.H.); fatima.heydari@monash.edu (F.H.); 2 Planet Innovation, Box Hill, VIC 3128, Australia; paul.howley@planetinnovation.com.au

**Keywords:** blind source separation (BBS), heart rate estimation, independent component analysis (ICA), ultra-wideband (UWB) radar

## Abstract

Vital signs such as heart rate and respiration rate are among the most important physiological signals for health monitoring and medical applications. Impulse radio (IR) ultra-wideband (UWB) radar becomes one of the essential sensors in non-contact vital signs detection. The heart pulse wave is easily corrupted by noise and respiration activity since the heartbeat signal has less power compared with the breathing signal and its harmonics. In this paper, a signal processing technique for a UWB radar system was developed to detect the heart rate and respiration rate. There are four main stages of signal processing: (1) clutter removal to reduce the static random noise from the environment; (2) independent component analysis (ICA) to do dimension reduction and remove noise; (3) using low-pass and high-pass filters to eliminate the out of band noise; (4) modified covariance method for spectrum estimation. Furthermore, higher harmonics of heart rate were used to estimate heart rate and minimize respiration interference. The experiments in this article contain different scenarios including bed angle, body position, as well as interference from the visitor near the bed and away from the bed. The results were compared with the ECG sensor and respiration belt. The average mean absolute error (MAE) of heart rate results is 1.32 for the proposed algorithm.

## 1. Introduction

Heart rate and respiration rate are two important factors of human’s body health. Resting heart rate can be a prognostic factor for coronary heart disease [1], and it highly relates to stroke, sudden death, and other non-cardiovascular diseases [2]. The traditional method to monitor heart rate is performed by using electrocardiography (ECG). However, it is not appropriate for long-time monitoring due to its limit of mobility and inconvenience. In addition, some people develop dermatitis after using ECG electrodes [3]. There is a demand for non-contact heart rate and respiration rate monitoring systems in hospitals. Camera-based heart rate monitoring technology is one of the popular research areas. L. Shan and M. Yu proposed an independent component analysis (ICA) and head tracking method to obtain the heart rate of subjects [4]. They used a face detector to determine a region of interest on the head. Then, applied band-pass filter (BPF) for both the x-axis and the y-axis. The heart rate result was displayed in fast Fourier transform (FFT) form after signal extraction through ICA. Patients may feel uncomfortable with the video-based monitoring systems because of the privacy problem. In [5], the authors used a light source and photodetector to obtain the chest movement and heartbeat signals. However, the results were affected by ambient light. A vibration-sensor-based monitoring system has been developed in [6,7] for sleep monitoring. In [7], they obtain the heart rate by using the autocorrelation function since the heart rate is a period signal, compared with the motion artifact. Researchers achieve multi-people heartbeat and respiration rate detection using the amplitude demodulation technique[7].

**Figure 1:**
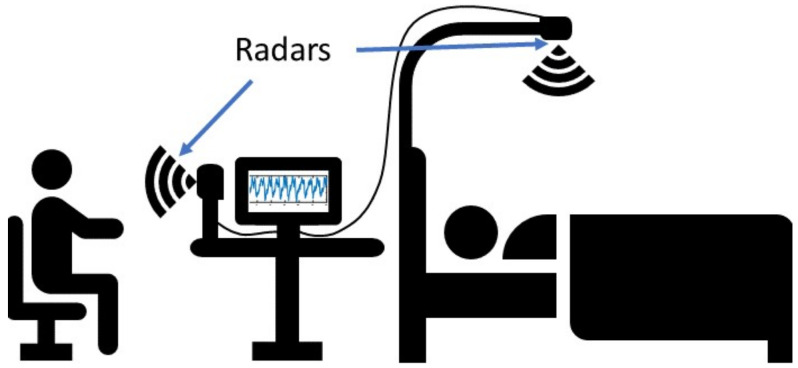
An overview of heart rate radar monitoring system.

There is also another non-contact vital signs monitoring technique using radar sensors [8,9,10,11,12,13,14,15,16,17,18,19,20,21,22,23,24,25,26]. The advantage of radar-based heart rate monitoring is that patients do not need to take off clothes and put on electrodes, which is convenient, and patients do not need to worry about privacy issues. Moreover, it can be used for both on-bed and off-bed situations, which is suitable for hospital application and 24 h monitoring. Figure 1 shows an overview of a radar-based heart rate monitoring system. Heart rate detection using a non-contact radar sensor is challenging because of the lower signal-to-noise ratio (SNR), compared with a contact-based heart rate monitor devices, especially when the range increases. Because radio frequency (RF) wave will experience path loss when it travels in the air, and it will lose power when RF wave penetrates through the body. This will cause low received signal power and low SNR. Moreover, the energy of the heart rate signal is lower, compared with energy in the respiration signal, and the respiration harmonics will cause interference to the heartbeat signal. Thus, it is a fundamental problem to detect signals in low SNR scenarios [27,28]. Self-motion and random body motion are other challenges in vital sign radar detection. Researchers in [29] use autocorrelation for phase and cross correlation for range bin to distinguish between stationary objects and a human to extract respiration rate. A phase average method for multiple frequencies is proposed in [30] to track the body motion of the test subject. In [11], Mostafanezhad et al. applied empirical mode decomposition (EMD) to remove the motion from a continuous wave (CW) radar signal and increase the accuracy of heart rate measurement. A least mean squares (LMS) adaptive filter was applied to the CW radar signal to do the respiration harmonics cancellation in [12]. Researchers in [13] used an artificial neural network (ANN) to extract a heart rate signal and detect heartbeat events with low latency and low computational complexity.

CW doppler radars are commonly used in vital signs detection [9,10]. Frequency modulated continuous wave (FMCW) radar is also employed in heart rate and respiration rate monitoring [14]. Moreover, biomedical multiple-input–multiple-output (MIMO) radars have been used for vital signs detection and human localization [26]. Compared with CW radar, FMCW radar can provide more information such as range, velocity, and even angle estimation [31]. Since FMCW radar has a range profile, detection of multiple subjects or people is possible [15,16]. Another common radar for vital sign monitoring is IR-UWB. It can also provide a range of information. The study in [18] used IR-UWB radar to monitor the respiration of people with dementia. IR-UWB radar has also been used in car applications [19]. Study [17] has shown that IR-UWB radar has higher SNR, simpler hardware structure, and better accuracy ratio for most scenarios, compared with FMCW radar. IR-UWB radar was used in this research as it provides higher SNR and accuracy.

There are different signal processing algorithms in radar-based heart rate detection. The blind source separation (BSS) method can extract and recover the signals from a mixture of noise and wanted signals. It has been used as a dimension reduction and signals separation technique in a vital signs radar system. In [20], M. Le proposed an eigenvalue-based method to extract and reconstruct respiration and heartbeat signals. Principal component analysis (PCA) was applied in [21] to remove the static noise and recover vital signals [22]. Two CW radars systems in [23] apply ICA to attenuate the breathing effect for heartbeat signal recovering. In [32] ICA-JADE and direction of arrival techniques were used to obtain the respiration rate of multiple people. The main signal processing techniques in the literature such as PCA, ICA, and eigenvalue-based methods belong to the BSS method.

Considering the advantages of IR-UWB radar in providing range information and higher SNR, compared with FMCW or CW radars, this study developed a signal processing approach for heart rate and respiration rate detection based on IR-UWB technology. ICA was applied as a BSS method in signal processing for dimension reduction, noise cancellation, and signal separation. In addition, a new method of signal processing based on higher-order harmonics peak selection was applied to successfully achieve a highly accurate heart rate. Comparing with the existing studies, this work provides a less complicated computation for signal separation, accurate heart rate detection, and it avoids the respiration harmonics interference from heart rate detection. The structure of this paper is as follows: Section 2 derives vital signal modelling. Section 3 discusses the signal processing algorithm in this paper. Section 4 presents the results of the experiments. Section 5 presents the conclusions.

## 2. Radar Signal Modelling

The content of this section is about modelling the IR-UWB radar signal for vital signs detection. In [8,24], the authors derived mathematical equations for the IR-UWB radar signal in detail. To model the equation of vital signs in IR-UWB radar, the authors assumed that the chest movement and heartbeat are periodic sinusoidal. The UWB radar transmits pulses in a short time. The received pulses represent signals in different distances according to the time of arrival (TOA). Then, the received signal for one channel can be written as

(1) $$d\left(t\right)=d_{0}+d_{r}sin\left(2\pi f_{r}t\right)+d_{h}sin\left(2\pi f_{h}t\right),$$

where *t* is slow time along the measurement interval; $d_{0}$ is the distance from the radar to the person; $d_{h}$ and $d_{r}$ are the amplitudes of heartbeat and respiration, respectively. The $f_{h}$ and $f_{r}$ parameters are the frequencies of heartbeat and respiration, respectively, [24].

The received pulse signal can be expressed as multipath components plus the reflected signal from the body as follows:

(2) $$r\left(t,\tau \right)=\sum_{i}A_{i}p\left(\tau -\tau_{i}\right)+Ap\left(\tau -\tau_{d}\left(t\right)\right),$$

where Ap is the amplitude of pulse at the target distance, τ is the time of arrival related to d(t), p(t) is received pulse in pass-band frequency, $A_{i}$ is the amplitude of multipath components and $\tau_{i}$ is the delay of a multipath signal. The time τ along to the range is fast time, and the time between each sample frame for all ranges is slow time. Since TOA is the time that a pulse transmits and receives, TOA from the person can be written as (3) [24]

(3) $$\tau_{d}\left(t\right)=\frac{2d(t)}{c}=\tau_{0}+\tau_{r}sin\left(2\pi f_{r}t\right)+\tau_{h}sin\left(2\pi f_{h}t\right),$$

where $\tau_{0}$ is the delay from the person, and $\tau_{r}$ and $\tau_{h}$ are the displacement of chest movement and heartbeat, respectively [24].

In (3), two terms of the radar signal model are corresponding to multipath components and vital signals. The multipath components derive from a static environment, which causes DC to offset in the received signal. To extract heartbeat and respiration signals and compensate DC offset, the received signal is subtracted by the average of fast–time channels, as (4) [8]

(4) $$y\left(t,\tau \right)=Ap(\tau -\tau_{d}\left(t\right)).$$

## 3. Signal Processing

**Figure 2:**
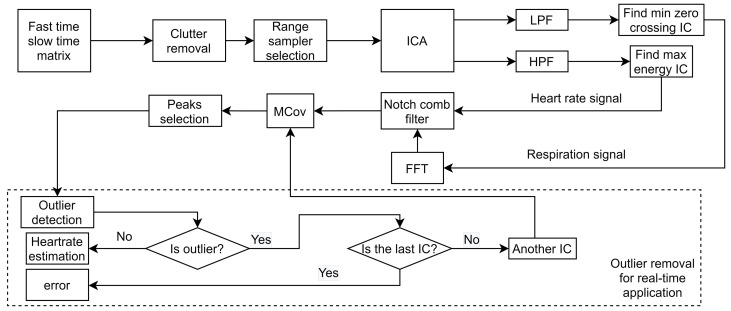
The block diagram of the signal processing algorithm.

Figure 2 shows the block diagram of the suggested signal processing approach in this study for heart rate and respiration rate detection using the IR-UWB radar. The input signal in the algorithm is a fast-time–slow-time matrix, which is the raw signal from the IR-UWB radar.

### 3.1. Clutter Reduction

A clutter reduction technique was applied to the raw signal to remove background noise [33]. Assuming a person is keeping still, clutter would be a stationary noise; therefore, it could be removed by the background subtraction method. Suppose that r(n) is the received signal of one range index; then, the clutter removal method can be written as (5) [33]:

(5) y(n)=r(n)−b(n−1),

where b(n−1) is the background clutter estimator. The stationary background clutter is defined as the average of previous samples (6) [33]

(6) $$b\left(n-1\right)=\frac{1}{M}\sum_{j-n-M}^{n-1}r\left(j\right).$$

### 3.2. Range Selection

The next step was to select the distance range in which the target is located. Assume that there is only one person in the radar detection range, and the person keeps still. Hence, the respiratory motion will have the largest energy in the received signals. The range selection can be conducted by choosing the maximum variance along with the slow-time of the range samplers, which contains a respiration signal. It is important to keep the nearby range samplers to cover cardiac activity since the breathing signal may not contain a heartbeat signal. In this experiment, the range bin of interest for the radar sensor was set to 60 cm to cover from the chest to the back of the body.

### 3.3. Independent Component Analysis

The purpose of this step was to recover the heart rate and respiration signals from a mixed signal and reduce the noise using the BSS method. The observation (received signal) for each range can be considered as the linear combination of various signals, e.g., cardiac signal, chest motion, and noise signal. Thus, the observation for each range can be written as follows:    

(7) x1(t)=a11s1(t)+a12s2(t)+…+a1nsn(t),x2(t)=a21s1(t)+a22s2(t)+…+a2nsn(t),x3(t)=a31s1(t)+a32s2(t)+…+a3nsn(t),b=⋮xn(t)=an1s1(t)+an2s2(t)+…+annsn(t),

where $x_{1}$, $x_{2}$ … $x_{n}$ are the observation signals in each range sampler index; $s_{1}$, $s_{2}$… $s_{n}$ are the original unknown signals, and $a_{ij}$ are the constant coefficients (weights) of each unknown signals. Equation (7) can be written in a matrix form as (8), [34].

(8) x=As,

where

(9) $$A=\left[\begin{array}{cccc} a_{11} & a_{12} & \ldots & a_{1n}\\ a_{21} & a_{22} & \ldots & a_{2n}\\ \ldots & \ldots & \ldots & \ldots \\ a_{n1} & a_{n2} & \ldots & a_{nn} \end{array}\right],x=\left[\begin{array}{c} x_{1}\left(t\right)\\ x_{2}\left(t\right)\\ \ldots \\ x_{n}\left(t\right) \end{array}\right]s=\left[\begin{array}{c} s_{1}\left(t\right)\\ s_{2}\left(t\right)\\ \ldots \\ s_{n}\left(t\right) \end{array}\right].$$

It is assumed that the signals $s_{i}$ are statistically independent and have non-Gaussian distributions, and the observation is a random vector x. To recover vital signals $s_{i}$, matrix A should be estimated first. If W is defined as the inverse matrix of A, then the independent components (IC) *s* can be computed as (10) [35].

(10) s=Wx.

Defining y as an IC, where column vector w is a row of the inverse of A, *y* can be calculated as (11) [34].

(11) $$y=w^{T}x=\sum_{i}w_{i}x_{i}.$$

Denote $z=A^{T}w$, then (11) can be written as (12) [34],

(12) $$y=w^{T}x=z^{T}s.$$

From (12), it is clear that *y* is the linear transformation of $s_{i}$, where the weights are vector *z*. Since the central limit theorem tells that the summation of independent random variables will become more Gaussian than any one of the original variables, the least Gaussianity will happen when $z^{T}s$ is equal to the one of IC $s_{i}$, and there is only one nonzero in weight *z*. Hence, w can be estimated by maximizing the non-Gaussianity of $w^{T}x$ [34].

There are different algorithms for ICA. The algorithm used in this paper was FastICA since it has low computation and uses a nonlinear algorithm that is robust [35]. The first step of ICA is centring data, which is *x* subtract the mean of itself. This can simplify the further process in the ICA algorithm.

(13) $$\hat{x}=x-E\left\{x\right\}.$$

After centring data, the next step is the whitening process, which reduces the computational complexity of FastICA. This process is to uncorrelate signals and normalise variance. The covariance matrix will become an identity matrix after whitening. The whitening process is performed through eigenvalue decomposition (EVD). EVD of the covariance matrix of $\hat{x}$ is calculated as

(14) $$E\left\{\hat{x}{\hat{x}}^{T}\right\}={\mathrm{EDE}}^{T},$$

where E is the matrix of eigenvector, and D is eigenvalues in a diagonal matrix. The data after whitening becomes

(15) $$\tilde{x}={\mathrm{ED}}^{-1/2}E^{T}\hat{x}={\mathrm{ED}}^{-1/2}E^{T}\mathrm{As}=\tilde{A}s,\;$$

where $\tilde{A}$ is the whitening transformation matrix.

The FastICA algorithm is using fixed-point arithmetic to solve the multi-dimension signals problem. The process of FastICA is described as Algorithm 1 [34].
**Algorithm 1** FastICA1:Choose the initial value of *w*.2:$w^{+}=E\left\{xg\left(w^{T}x\right)\right\}-E\left\{g^{\prime }\left(w^{T}x\right)\right\}w$3:$w=w^{+}/∥w^{+}∥$4:**if***w* is not converged **then**5:    go to 26:**end if**where function *g* is
(16)$$\begin{array}{cc} g_{1} & =tanh(a_{1}y),\\ g_{2} & =yexp(-\frac{y^{T}}{2}),\\ g_{3} & =y^{3}. \end{array}$$$a_{1}$ is a constant 1 < $a_{1}$ < 2

### 3.4. Modified Covariance Method

All ICs have the same energy because the variances of ICs are unit. To obtain the respiration rate, ICs were filtered using a low-pass filter (LPF). Since the respiration rate at rest for healthy people is between 12 bpm to 20 bpm [36], the cutoff frequency of the LPF was chosen as 0.8 Hz (48 bpm). Considering that the respiration signal has less zero-crossing, compared to the heart rate signal, the ICs after LPF were compared, and the one with the least zero-crossing was chosen for respiration detection. The FFT was applied to the selected IC, and the frequency component with the maximum power was considered as respiration rate.

For the heart rate estimation, the ICs were passed through a high-pass filter (HPF) separately, as shown in Figure 2. The cutoff frequency of the HPF was chosen as 1.66 Hz to eliminate the frequency components related to respiration and its strongest harmonics. The energy of heart rate harmonics is more than the energy of respiration harmonics in the heart rate harmonics related frequency ranges [8]; therefore, a comparison was made between the energy of ICs after HPF, and the maximum energy IC was selected as the heart rate signal.

The measured breathing rate was used as a parameter for a notched comb filter to reduce the respiration harmonics’ effect on heart rate estimation. After notched filter, the modified covariance method was used as the spectrum estimation. The modified covariance is an autoregressive (AR) spectrum estimation method. The benefit of the modified covariance method is that it results in stable spectrum estimation with minimised forward and backward prediction errors. The AR coefficients can be calculated by solving (17) [37].

(17) $$\left[\begin{array}{cccc} r_{x}\left(1,1\right) & r_{x}\left(2,1\right) & \ldots & r_{x}\left(p,1\right)\\ r_{x}\left(1,2\right) & r_{x}\left(2,2\right) & \ldots & r_{x}\left(n,2\right)\\ \ldots & \ldots & \ldots & \ldots \\ r_{x}\left(1,p\right) & r_{x}\left(2,p\right) & \ldots & r_{x}\left(p,p\right) \end{array}\right]\left[\begin{array}{c} a_{p}\left(1\right)\\ a_{p}\left(2\right)\\ \ldots \\ a_{p}\left(p\right) \end{array}\right]=-\left[\begin{array}{c} r_{x}\left(0,1\right)\\ r_{x}\left(0,2\right)\\ \ldots \\ r_{x}\left(0,p\right) \end{array}\right],$$

where

(18) $$\begin{array}{cc} r_{x}\left(k,l\right)= & \sum_{n=p}^{N-1}[x\left(n-l\right)x^{*}\left(n-k\right)\\ \; & +x\left(n-p+l\right)x^{*}\left(n-p+k\right)]. \end{array}$$

In (17) and (18), $r_{x}$ is autocorrelation sequence of *x* and $a_{p}$ is coefficient of the poles. The power spectrum density (PSD) estimation of the AR process is as (19) [37].

(19) $${\hat{P}}_{AR}\left(e^{j}\omega \right)=\frac{|\hat{b}{\left(0\right)|}^{2}}{|1+\sum_{k=1}^{p}{\hat{a}}_{p}\left(k\right)e^{-j\omega k}{|}^{2}},$$

where

(20) $${\left|b\left(0\right)\right|}^{2}=r_{x}\left(0,0\right)+\sum_{k=1}^{p}a_{p}\left(k\right)r_{x}^{*}\left(0,k\right).$$

In (19), *b* is the coefficient of zero in AR model.

### 3.5. Peak Selection Algorithm

Because the heart rate in the frequency domain is easily contaminated by respiration and its harmonics, it is hard to obtain an accurate heart rate using the modified covariance method. Therefore, this study proposed a peak selection method based on high harmonics of heart rate.

First, denote that array *P* has the elements $p_{1}$, $p_{2}$…$p_{n}$, which are all peaks above 100 bpm.

Then, the algorithm finds all peaks $p_{i}$ between 100 bpm and 400 bpm, and puts all their related frequencies in a vector, as (21).

(21) $$P=\left[\begin{array}{cccc} p_{1} & p_{2} & \ldots & p_{n} \end{array}\right].$$

The next step is finding two initial values for heart rate, as (22).

(22) $$HR_{guess}=\left[\begin{array}{cc} hr_{guess1} & hr_{guess2} \end{array}\right].$$

If the difference between $p_{1}$ and $p_{2}$ is less than 100 bpm, $p_{1}$ and $p_{2}$ are considered as the first two harmonics of the heart rate and divided by 2 and 3, respectively, as the heart rate initial assumptions $hr_{guess1}$ and $hr_{guess2}$. If the difference between $p_{1}$ and $p_{2}$ is greater than or equal to 100 bpm, $p_{1}$ is considered as the initial fundamental value for the heart rate $hr_{guess1}$, and $p_{2}$ is assumed as the second harmonic and divided by 2 to obtain the $hr_{guess2}$.

Next, vector *P* is divided by $hr_{guess1}$ and $hr_{guess2}$ separately and rounded to obtain the integer multiple arrays $Int1_{mul}$ and $Int2_{mul}$, as (23) and (24).

(23) $$\begin{array}{cc} Int_{mul} & =round\left(\left[P^{T}P^{T}\right]\times \left[\begin{array}{cc} \frac{1}{hr_{guess1}} & 0\\ 0 & \frac{1}{hr_{guess2}} \end{array}\right]\right)=\left[\begin{array}{cc} int1_{mul} & int2_{mul} \end{array}\right], \end{array}$$

where

(24) $$\begin{array}{c} int1_{mul}={\left[\begin{array}{cccc} int1_{mul1} & int1_{mul2} & \ldots & int1_{muln} \end{array}\right]}^{T},\\ int2_{mul}={\left[\begin{array}{cccc} int2_{mul1} & int2_{mul2} & \ldots & int2_{muln} \end{array}\right]}^{T}. \end{array}$$

After that, *P* is element-wise divided by $Int1_{mul}$ and $Int2_{mul}$ separately to obtain the heart rate estimation array $hr1_{est}$ and $hr2_{est}$, as (25) and (26).

(25) $$hr_{est}=\left[\begin{array}{cc} P^{T} & P^{T} \end{array}\right]./\left[\begin{array}{cc} int1_{mul} & int2_{mul} \end{array}\right]=\left[\begin{array}{cc} hr1_{est} & hr2_{est} \end{array}\right],$$

where

(26) $$\begin{array}{c} hr1_{est}=\left[\begin{array}{cccc} hr1_{est1} & hr1_{est2} & \ldots & hr1_{estn} \end{array}\right],\\ hr2_{est}=\left[\begin{array}{cccc} hr2_{est1} & hr2_{est2} & \ldots & hr2_{estn} \end{array}\right]. \end{array}$$

Later, the error arrays error1 and error2 are calculated by subtracting $hr_{guess1}$ and $hr_{guess2}$ from $hr1_{est}$ and $hr2_{est}$, respectively, as (27) and (28).

(27) $$Error=abs\left(\left[\begin{array}{cc} hr1_{est} & hr2_{est} \end{array}\right]-\left[\begin{array}{cc} hr_{guess1} & hr_{guess2} \end{array}\right]\right)=\left[\begin{array}{cc} error1 & error2 \end{array}\right],$$

where

(28) $$\begin{array}{c} error1=\left[\begin{array}{cccc} error1_{1} & error1_{2} & \ldots & error1_{n} \end{array}\right],\\ error2=\left[\begin{array}{cccc} error2_{1} & error2_{2} & \ldots & error2_{n} \end{array}\right]. \end{array}$$

If the calculated errors are above a threshold (6 bpm), consider the corresponding high-frequency peak as a noise. Remove estimated heart rates and errors according to its index in the heart rate estimation array.

Finally, the averages of the remaining errors for error1 and error2 are calculated, and the corresponding estimated heart rate array to the minimum average value is selected, $hr_{slt}$. The final heart rate $hr_{est}$ will be calculated as the average of the $hr_{slt}$, as (29).

(29) $$hr_{est}=mean\left(hr_{slt}\right),$$

where

(30) $$hr_{slt}=\begin{array}{cc} hr1_{est}, & \mathrm{if}\;mean(error1)\leq mean(error2)\\ hr2_{est}, & \mathrm{otherwise}. \end{array}$$

### 3.6. Outliers Removal in Real-Time Measurement

The proposed algorithm can be implemented for a real-time application. The window length for each reading is considered 35 s, and the readings are repeated every 5 s. Hence, the program waits for 35 s after the sensor starts for the first reading. Algorithm 2 shows the process of outliers removal. The first five estimations for heart rate are stored in a first-in-first-out (FIFO) buffer. Starting from the sixth measurement, the program compares the new heart rate estimation with the FIFO buffer’s median value.
**Algorithm 2**Outliers removal1:Put the first five results into FIFO buffer2:**if**$hr_{est}$ —median(FIFO) < threshold **then**3:    go to line 174:    error_count = 0;5:**else**6:    **if** is last IC **then**7:        error_count = error_count + 1;8:        discard result9:    **else**10:        select another IC to do peak selection11:        go to line 212:    **end if**13:**end if**14:**if**error_count = 5 **then**15:      reset FIFO and go to 116:**end if**17:returns result.

Suppose the difference between the FIFO buffer’s median value and the latest reading is less than the threshold (10% of median value). In that case, the new result will be considered as the estimated heart rate and updated to the FIFO buffer. Otherwise, the program takes another IC to repeat the peak selection algorithm and achieve the results. It will continue changing the IC until the last estimated heart rate is within the threshold. If none of the ICs gives the correct estimation, the program discards the estimated value.

## 4. Experiment Results and Discussion

### 4.1. Experiment Setup

The UWB radar used in this paper was Xethru X4M200 [38,39]. It can operate in the low-frequency band and high-frequency band. The detection range is also configurable from 0.4 m to 5 m with a resolution of 0.0514 m [38,39]. Table 1 illustrates the configuration of the Xethru radar set for this study. The radar sensor was connected to the laptop with a MATLAB API interface to collect raw data. Meanwhile, an ECG sensor and a respiration belt were connected to another laptop as reference signals.

**Figure 3:**
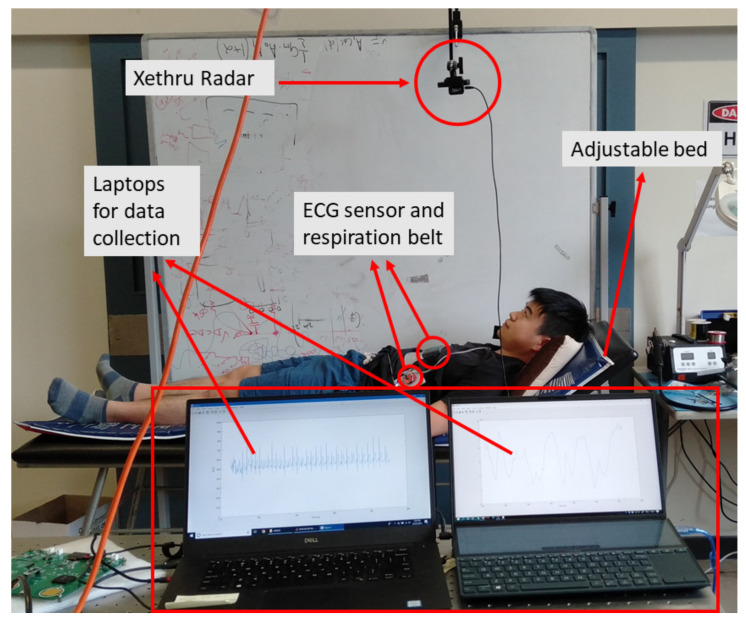
The setup of devices: the radar is mounted 1 m above the bed. Two laptops are used for radar sensor data and reference signals recording.

To evaluate the reliability of the algorithm for heart rate and respiration rate detection, a data collection was performed which covers different scenarios of a person on a bed. The details of the suggested protocols for the experiments are explained in Table 2. The data from five healthy participants were collected. None of them reported any cardiovascular or respiratory system disorders. Figure 3 shows the setup of devices during the experiment. Xethru was mounted 1 m above the bed pointing to the chest of the subject.

### 4.2. Results and Discussion

**Figure 4:**
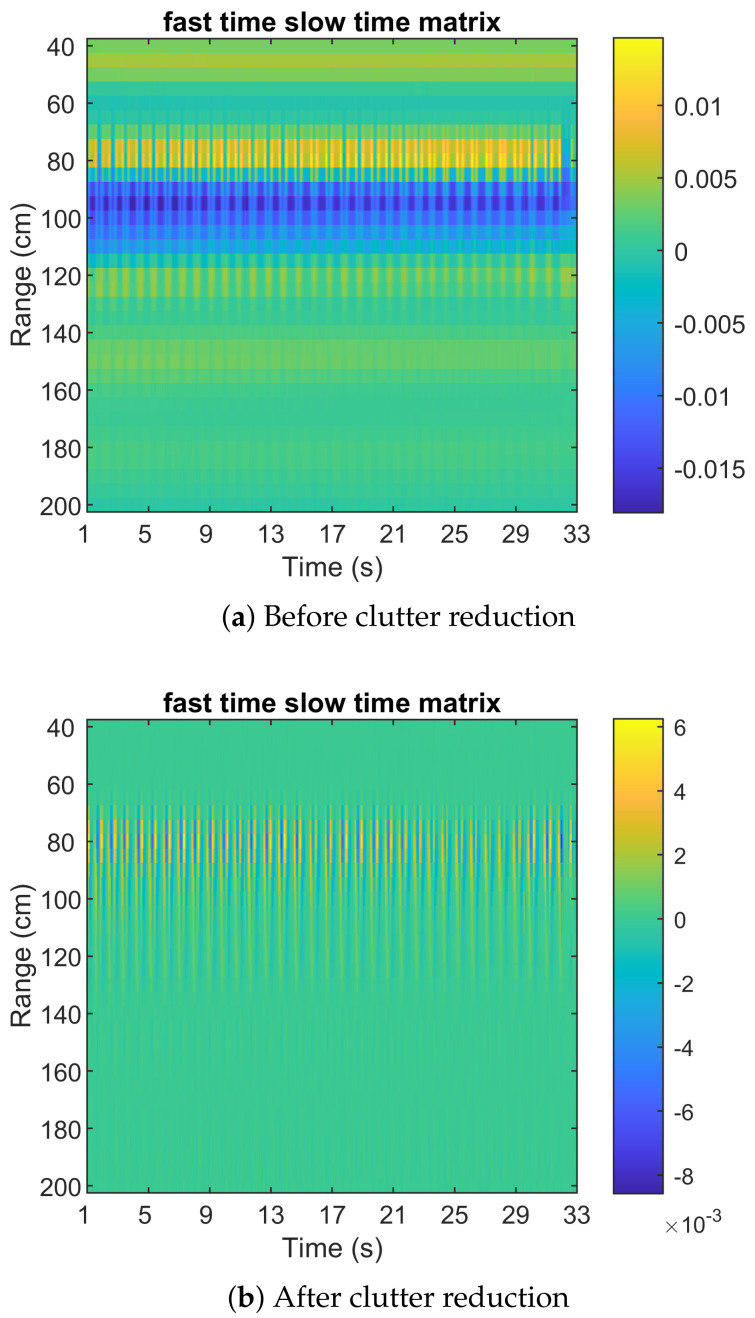
Fast–time–slow–time matrix.

Figure 4a shows the fast time and slow time of the raw signal, and Figure 4b illustrates the result after clutter reduction based on the background subtraction method. In Figure 4b, the maximum energy is around 80 cm.

**Figure 5:**
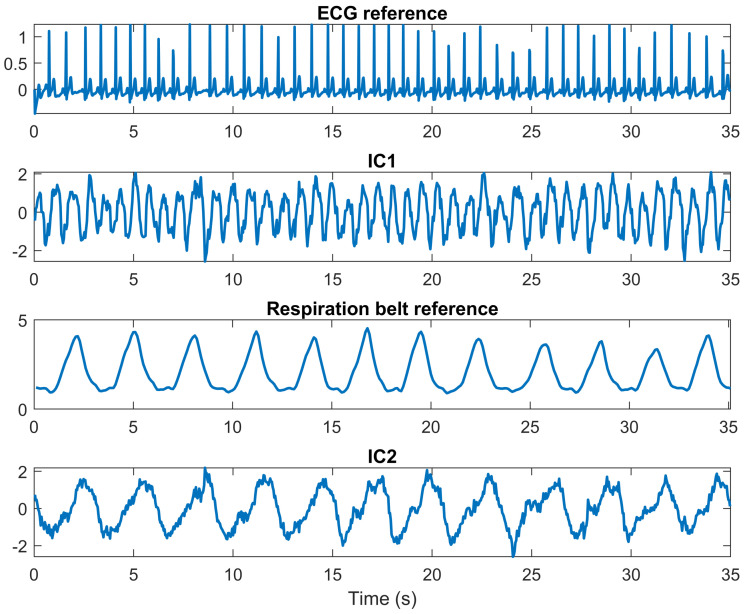
ECG, respiration signals, and ICs.

**Figure 6:**
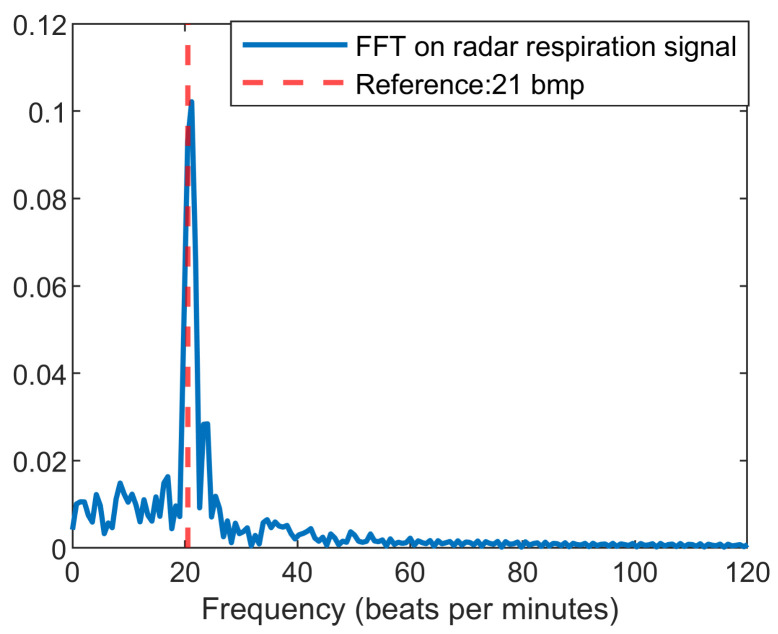
Result of FFT on extracted breathing signal.

Figure 5 indicates a fast-time–slow-time matrix after ICA and reference signals. It is clear that after vital signals extraction, IC1 is the heartbeat signal, whereas IC2 is the chest cavity motion during breathing. Figure 6 illustrates the FFT result of IC2 in Figure 5 for respiration rate detection. The peak of FFT is represented as the respiration rate value with high accuracy.

**Figure 7:**
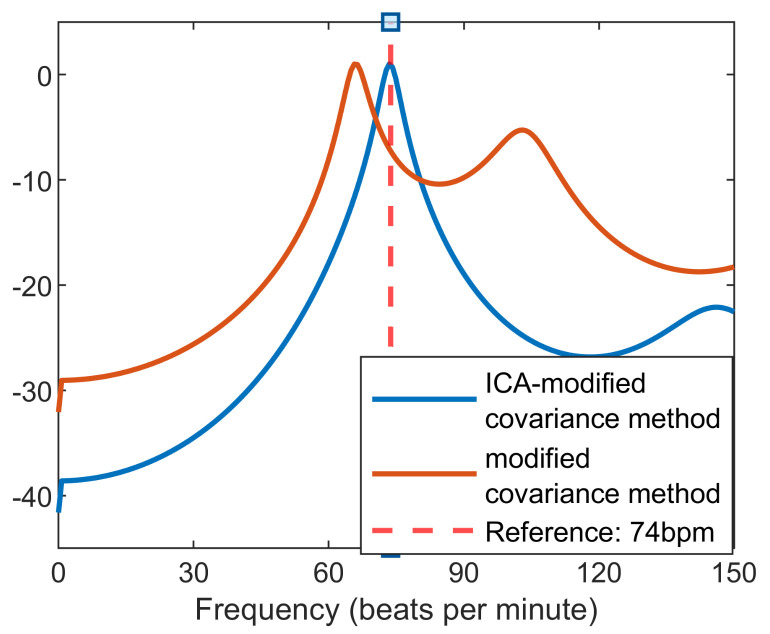
Results of modified covariance method before ICA vital signs extraction and after vital signs extraction.

**Figure 8:**
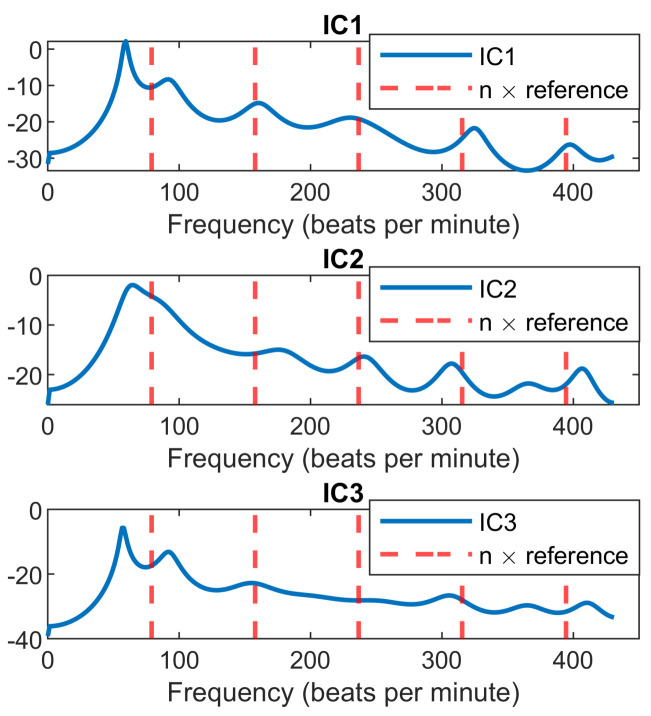
Results of modified covariance method for all ICs. The reference heart rate is 79 bpm. The red dash lines are the integer multiple of reference heart rate.

Figure 7 clearly shows that after heartbeat signal extraction by ICA, the peak of PSD is closer to the reference heart rate, compared with the derived heart rate after high-pass filtering. Using ICA and a simple peak detection algorithm to find the accurate peak of a signal corresponding to the correct heart rate is not practical most of the time. Figure 8 illustrates an example of a situation where the fundamental heart rate is corrupted by respiration harmonics. It shows that all ICs have the highest peak around 60 bpm, whilst the reference heart rate value is around 79 bpm. Moreover, the harmonics of heart rate are almost aligned with the integer multiple of reference. This is the reason the proposed peak selection algorithm always works with the higher harmonics for heart rate extraction.

**Figure 9:**
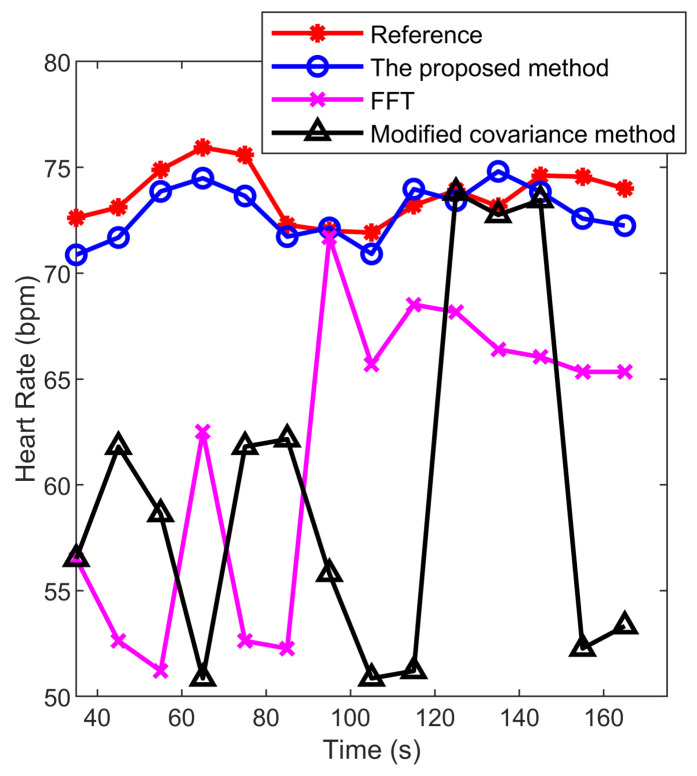
Heart rate estimation with different methods with a window length of 35 s. The red star is the reference heart rate, the pink cross is FFT after the high-pass filter, the black triangle is the modified covariance method after the high-pass filter, and the blue circle is the proposed method.

Figure 9 indicates the extracted heart rate using different algorithms and the reference heart rate values from ECG. The window length for each radar and ECG signals’ reading is 35 s, and the readings are repeated every 5 s. The figure shows that the FFT and modified covariance method are affected by respiration harmonics and can not achieve an accurate result. The proposed peak detection algorithm has higher accuracy and stability, compared with other methods.

Table 3 presents the estimated heart rates and respiration rates of one subject’s data for the previously mentioned scenarios. The error in this table is calculated using the absolute value of the difference between the reference ground truth and the measurement. It shows that the average error is 0.82 bpm for the respiration signal, and the maximum error is 2 bpm. In contrast, for heart rate estimation, the average error is 1.45 bpm. The maximum error is 4 bpm for Fowler’s position 2 and the visitor sitting near the head of the subject. Table 3 also indicates that different body positions and a visitor away 0.5 m or 1 m from the subject will give more accurate results for both heart rate and respiration rate estimation.

The results of the heart rates and respiration rates are displayed in Table 4. The mean absolute error (MAE) and root mean square error (RMSE) for all five subjects who participated in the experiments are compared with their related reference signals. It is clear that the average MAE is 0.65 for respiration rate and 1.32 for heart rate estimation in Table 4. The maximum MAE for heart rate happens in the scenarios of left lateral recumbent and the visitor sitting and talking near the leg of the subject. It is because the received signal will be weak when the radar signal penetrates from the right arm to the heart of the subject, and the visitor talking interferes with the heartbeat signal received from the radar sensor. Moreover, the heart rate detection will be accurate when a visitor is 0.5 m and 1 m away from the subject on the bed. Table 5 shows a comparison between the devices and signal processing approaches from different studies and the proposed method in this work.

(31) $$MAE=\frac{\sum_{i=1}^{n}\left|y_{i}-x_{i}\right|}{n}=\frac{\sum_{i=1}^{n}e_{i}}{n},$$

(32) $$RMSE=\sqrt{\frac{1}{n}\sum_{i=1}^{n}{\left(y_{i}-x_{i}\right)}^{2}}.$$

In the Equations $y_{i}$ is the measurement and $x_{i}$ is the ground truth of reference signals.

## 5. Conclusions

In this paper, we proposed a novel signal processing algorithm for heart rate and respiration rate estimation based on the ICA and harmonics peak selection method for an IR-UWB radar sensor. The suggested algorithm used ICA to extract clear pulse waves and respiratory signals. Then, the modified covariance method was used to obtain the frequency domain PSD after filtering. Since the fundamental heartbeat signal is easily corrupted by the respiratory signal and its harmonics, a peak selection based on higher harmonics of the heart waveform was introduced to estimate the heart rate. The outliers detection algorithm was applied to improve the accuracy of the results further in the real-time application because of its capability to consider other ICs if there is an outlier in the detected heart rates. The results were measured with five subjects with different situations in this experiment. The result gives an MAE of 0.65 in respiration and 1.32 in heart rate detection.

There are some limitations to the proposed algorithm. The signal is easily interfered with by vibration noise from the environment with a frequency close to the heart rate because the energy of heart rate harmonics is low. In the future, our work will focus on multiple people detection, localization, heart rate, respiration rate detection in noisy environments such as body movement, talking, and walking scenarios.

## Figures and Tables

**Table 1:** UWB radar parameters.

Parameters	Values
Centre frequency	7.29 GHz
Bandwidth	1.4 GHz
ADC sampling rate	23.328 GS/s
Frame rate	24 fps
Detection range	0.4–5 m
Range resolution	0.0514 m

**Table 2:** Data collection protocol.

	Scenarios	Time
body position	Supine	3 min
	Right lateral recumbent	3 min
	Left lateral recumbent	3 min
	Prone	3 min
	Baby position (curled up) right	3 min
	Baby position (curled up) left	3 min
Different bed angle	Fowler’s position 1 (20 degree)	3 min
	Fowler’s position 2 (40 degree)	3 min
	Fowler’s position 3 (55 degree)	3 min
	Sitting on the bed (90 degree)	3 min
Visitor near bed	Visitor standing near head	3 min
	Visitor sitting near head	3 min
	Visitor sitting and talking near head	3 min
	Visitor standing near leg	3 min
	Visitor sitting near leg	3 min
	Visitor sitting and talking near leg	3 min
Visitor far from bed	Visitor 0.5 m near head of bed	3 min
	Visitor 1 m near head of bed	3 min
	Visitor 0.5 m near leg	3 min
	Visitor 1 m near leg	3 min
	Visitor 0.5 m near tail of bed	3 min
	Visitor 1 m near tail of bed	3 min

**Table 3:** Results of heart rate and respiration rate in different scenarios. The data length is 35 s in this table for each experiment.

Scenarios	Respiration Rate			Heart Rate		
	Reference	Radar	Error	Reference	Radar	Error
Supine	21	20	1	74	75	1
Right lateral recumbent	18	18	0	76	76	0
Left lateral recumbent	19	19	0	71	72	1
Prone	18	18	0	71	70	1
Baby position right	18	19	1	68	67	1
Baby position left	18	19	1	68	67	1
Fowler’s position 1	21	20	1	71	70	1
Fowler’s position 2	18	20	2	70	66	4
Fowler’s position 3	20	20	0	72	75	3
Sitting on the bed	19	18	1	78	77	1
Visitor standing near head	18	19	1	70	68	2
Visitor sitting near head	19	21	2	69	65	4
Visitor sitting and talking near head	22	21	1	72	72	0
Visitor standing near leg	16	17	1	67	66	1
Visitor sitting near leg	17	18	1	65	63	2
Visitor sitting and talking near leg	18	20	2	65	65	2
Visitor 0.5 m near head of bed	20	20	0	77	78	1
Visitor 1 m near head of bed	18	19	1	71	74	3
Visitor 0.5 m near leg	19	19	0	68	68	0
Visitor 1 m near leg	18	18	0	67	66	1
Visitor 0.5 m near tail of bed	19	20	1	67	66	1
Visitor 1 m near tail of bed	19	18	1	67	66	1
Average error			0.82			1.45

**Table 4:** Results of different errors.

Scenarios	Respiration Rate		Heart Rate	
	MAE	RMSE	MAE	RMSE
Supine	1	1.18	1	2.1
Right lateral recumbent	0.2	0.45	1.4	1.95
Left lateral recumbent	0	0	2.6	4.171
Prone	0.2	0.45	2	2.61
Baby position right	0.8	1.1	1.4	1.95
Baby position left	0.6	1	2	2.61
Fowler’s position 1	1	1.18	1	1
Fowler’s position 2	0.8	1.1	2.2	2.49
Fowler’s position 3	0.4	0.632	1.2	1.67
Sitting on the bed	0.6	0.77	1.4	1.48
Visitor standing near head	0.4	0.63	1.6	1.79
Visitor sitting near head	0.6	0.77	0.8	1.1
Visitor sitting and talking near head	0.8	1.1	0.6	0.77
Visitor standing near leg	1	1.61	1	1.34
Visitor sitting near leg	1.2	1.26	0.8	1.1
Visitor sitting and talking near leg	1	1.34	2.6	2.93
Visitor 0.5 m near head of bed	0.6	0.77	2	2.68
Visitor 1 m near head of bed	0.4	0.89	0.6	0.77
Visitor 0.5 m near leg	1.4	1.48	0.6	1
Visitor 1 m near leg	0.6	0.77	0.8	1.41
Visitor 0.5 m near tail of bed	0	0	0.8	1.1
Visitor 1 m near tail of bed	0.8	1.1	0.6	0.77
Average error	0.65	0.89	1.32	1.76

**Table 5:** Comparison related work with the proposed method.

	Radar System	Radar Frequency (GHz)	Distance (m)	Position	Method
[9]	CW radar	24	0.75	Sitting	Estimate the coarse HR first, then use narrow BPF according to coarse HR.
[17]	UWB radar, FMCW radar	8.7	0.5–2.5	Front, left, right, back	MTI and FFT
[20]	UWB radar	4.6	0.5–3	Front, 45 degree, lateral side, backside	SVD and CZT
[23]	Two CW radars	10.587 and 10.525	0.5	Sitting	ICA and HPF
[25]	CW radar	26.4	0.6	Sitting	Filter only
Proposed method	UWB radar	7.29	1	Different bed angle and different body position	ICA, modified covariance method and peak selection

## Data Availability

Not applicable.
